# Comparison of implant quality between intraoperatively built custom-linked seeds and loose seeds in permanent prostate brachytherapy using sector analysis

**DOI:** 10.1093/jrr/rrw018

**Published:** 2016-08-03

**Authors:** Norihisa Katayama, Mitsuhiro Takemoto, Atsushi Takamoto, Hiroki Ihara, Kuniaki Katsui, Shin Ebara, Yasutomo Nasu, Susumu Kanazawa

**Affiliations:** 1 Department of Radiology, Okayama University Graduate School of Medicine, 2–5–1 Shikata-cho, Okayama 700–8558, Japan; 2 Department of Radiotherapy, Himeji Red Cross Hospital, Himeji, Japan; 3 Department of Urology, Okayama University Graduate School of Medicine, 2–5–1 Shikata-cho, Okayama 700–8558, Japan

**Keywords:** prostate cancer, brachytherapy, intraoperatively built custom-linked seeds, dosimetry, sector analysis, seed migration

## Abstract

We compared the implant quality of intraoperatively built custom-linked (IBCL) seeds with loose seeds in permanent prostate brachytherapy. Between June 2012 and January 2015, 64 consecutive prostate cancer patients underwent brachytherapy with IBCL seeds (*n* = 32) or loose seeds (*n* = 32). All the patients were treated with 144 Gy of brachytherapy alone. Brachytherapy was performed using a dynamic dose calculation technique. Computed tomography/magnetic resonance imaging fusion-based dosimetry was performed 1 month after brachytherapy. Post-implant dose–volume histogram (DVH) parameters, prostate sector dosimetry, operation time, seed migration, and toxicities were compared between the IBCL seed group and the loose seed group. A sector analysis tool was used to divide the prostate into six sectors (anterior and posterior sectors at the base, mid-gland, and apex). V100 (95.3% vs 89.7%; *P* = 0.014) and D90 (169.7 Gy vs 152.6 Gy; *P* = 0.013) in the anterior base sector were significantly higher in the IBCL seed group than in the loose seed group. The seed migration rate was significantly lower in the IBCL seed group than in the loose seed group (6% vs 66%; *P* < 0.001). Operation time per seed was significantly longer in the IBCL seed group than in the loose seed group (1.31 min vs 1.13 min; *P* = 0.003). Other post-implant DVH parameters and toxicities did not differ significantly between the two groups. Our study showed more dose coverage post-operatively in the anterior base prostate sector and less seed migration in IBCL seed implantation compared with loose seed implantation.

## INTRODUCTION

Permanent prostate brachytherapy (PPB) has become a standard treatment option for patients with localized prostate cancer, with long-term local and biochemical control similar to outcomes observed after radical prostatectomy and external beam radiation therapy (EBRT) [1–3]. In Japan, PPB was first adopted in 2003, and 109 institutions have used this treatment for 27 000 cases throughout the country up to the end of 2013 [4]. However, because stranded or linked seeds had not been introduced until 2012, only loose seeds had been used for PPB until then.

An intraoperatively built custom-linked (IBCL) seeds system is a push-button seed delivery system that allows the user to create intraoperatively customized linked seeds, using a combination of seeds, connectors and spacers. Zauls *et al*. first reported this system in 2010 [5]. It was introduced into Japan in 2012.

Sector analysis was developed by Bice *et al*., and is a method in which the organ is divided into different sectors according to anatomic locations [6]. Sector analysis of the prostate allows dose calculations not only to the whole prostate but also to specific parts of it [6–8].

To date, only three studies have compared the implant quality of IBCL seeds with that of loose seeds for use in PPB [5, 9, 10]. Although the three studies compared post-implant dose–volume histogram (DVH) parameters, seed migration rates, and operation times between the IBCL seed group and the loose seed group, they did not use sector analysis. Therefore, we compared the implant quality of IBCL seeds with that of loose seeds in PPB using sector analysis.

## MATERIALS AND METHODS

### Study design

This retrospective study was approved by the Institutional Review Board. Between June 2012 and January 2015, 64 consecutive patients with low-risk or intermediate-risk prostate cancer (prostate-specific antigen level ≤20 ng/ml; Gleason score 6–7; Union Internationale Contre le Cancer 2009 clinical stage T1–T2) were treated with PPB at Okayama University Hospital (Table 1). All patients were treated with brachytherapy alone using ^125^I radioactive seeds. IBCL and loose seeds were alternately used basically. All patients were treated by the same radiation oncologist (N.K.) and urologist (A.T.). Both had been well trained in PPB.

**Table 1.** Patient characteristics

	IBCL group^a^	Loose group^a^	*P* value
*n*	32	32	
Age (years)	66.3 ± 4.8	67.6 ± 6.3	0.11
PSA (ng/ml)	6.14 ± 2.09	7.41 ± 3.21	0.81
T stage (1c/2a/2b/2c)	15/9/5/3	18/11/2/1	0.43
Gleason score (3+3/3+4)	24/8	20/12	0.28
Hormonal therapy	20	21	0.79

^ a^Values are given as number or mean ± SD. IBCL = intraoperatively built custom-linked, PSA = prostate-specific antigen.

All patients underwent an ultrasonography volume study 2–6 weeks before implantation to determine the number of seeds to order. Loose seeds (Oncoseed; GE Healthcare, Medi-Physics, Arlington Heights, IL) were implanted using a Mick applicator (Mick Radio Nuclear Instruments, Mount Vernon, NY). The seed activity of the loose seed was 0.35 mCi/seed. The IBCL seeds were constructed using a Quicklink device (CR Bard, Covington, GA) and implanted. The seed activity of the IBCL seeds was 0.367 mCi/seed. Zauls *et al*. [5] have described the detailed procedure of constructing IBCL seeds, and we used a procedure similar to theirs. The radiation oncologist constructed the IBCL seeds.

Transrectal ultrasonography images in the axial plane were imported into the Variseed (Varian Medical Systems, Palo Alto, CA) brachytherapy planning system. The prostate, urethra, and rectal wall were contoured by the radiation oncologist. Needles were placed in the periphery of the gland 0.5–1 cm apart at the largest cross-section of the gland by the urologist. The radiation oncologist determined the positions of the centrally located needles. The planning software determined the first plan of seed number and location combinations for both the peripheral and centrally located needles. Modifications to the plan were made by the radiation oncologist, and the software recalculated the DVH and isodose lines in real time. The urologist placed the seeds, and the radiation oncologist performed a dynamic dose calculation [11]. For the IBCL seeds, the radiation oncologist both constructed the IBCL seeds and performed the dynamic dose calculation.

The prescribed dose was set at 144 Gy. Dose–volume targets were as follows: V100 (the percentage of the prostate volume that receives 100% of the prescribed dose) >99%; UD1 (the dose irradiating 1% of the urethral volume) <200 Gy; and RV100 (the rectal volume that receives 100% of the prescribed dose) <0.5 ml. We placed a few seeds inside the seminal vesicle, if necessary, but only with the IBCL seed. For both the IBCL and loose seed we placed some seeds outside the prostate capsule to achieve the urethral dose–volume target.

Post-implant dosimetry was performed using computed tomography (CT)/magnetic resonance imaging (MRI) fusion 1 month after implant. The same radiation oncologist (N.K.) performed the post-implant dosimetry for all patients, and the results were confirmed by the second radiation oncologist (M.T.). The second radiation oncologist (M.T.) had also been well trained in PPB. No urinary catheter was put in place. A CT scan was obtained using a 128-channel, dual-source multidetector-row CT scanner (SOMATOM Definition Flash, Siemens AG, Forchheim, Germany). Axial 0.6-mm-thick CT images of the prostate at 0.6-mm intervals were obtained. For these scans, a field of view (FOV) of 15 cm and a 512-square matrix were used. An MRI scan was obtained immediately after the CT using a 3-tesla (T) MRI scanner (Magnetom Verio 3 T, Siemens AG, Forchheim, Germany). Axial T2-weighted images of the prostate were obtained using a 16-channel body array anterior and posterior coil. Technical parameters were as follows: repetition time (TR)/echo time (TE) in milliseconds, 4000/99; FOV, 20 cm; slice thickness, 3 mm without a gap; matrix size, 224 × 320. The CT and MRI were electronically fused using the manual-fusion procedure of the Variseed fusion system. We identified nine or more corresponding seed pairs in the CT and MRI and then used the Variseed program to calculate the transformation. This fusion procedure was performed until the positions of the seeds in the CT image corresponded with those in the MRI. On CT, the seeds were detected automatically using the Variseed system, and the rectum was manually contoured. On MRI, the prostate and urethra were manually contoured. The urethra and rectum were contoured using the same slices as the prostate contour.

**Fig. 1.**
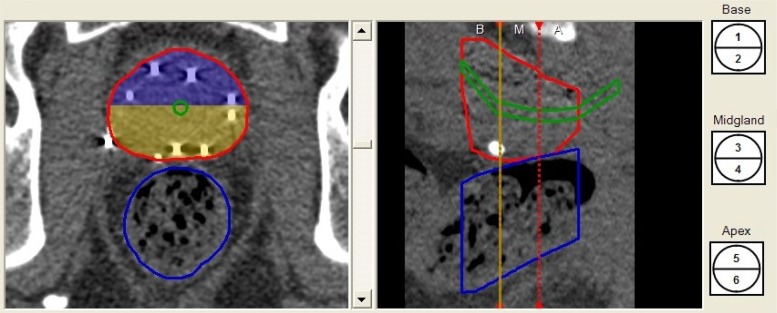
The sector analysis tool was used to divide the prostate into six sectors: anterior and posterior sectors at the base, mid-gland, and apex.

A sector analysis tool was used to divide the contoured prostate into six sectors: anterior and posterior sectors at the base, mid-gland, and apex (Fig. 1). Evaluation of radiation coverage in each of the six sectors was performed for both the intraoperative plan and the post-implant dosimetry. V100 and D90, the dose irradiating 90% of the prostate volume, were calculated and compared between the two groups.

On the same day as the CT/MRI, a chest radiograph (anteroposterior view) and an abdominal radiograph were taken to check for any possible seed migration. Seed migration was defined as a seed separated from the main seed cluster. The definition of ‘distal seed migration’ was one or more seeds located distally toward the perineum due to needle drag.

The urinary and rectal toxicities were evaluated in accordance with the Japanese version of the National Cancer Institute's Common Terminology Criteria for Adverse Events, version 4.0.

### Statistical analysis

We compared the baseline characteristics and the outcomes of interest between the IBCL seed group and the loose seed group using the 2-sample *t* test for continuous data that followed a normal distribution, the Mann–Whitney test for continuous data that did not follow a normal distribution, and the Chi-squared test for categorical data. Probability (*P*) values of <0.05 were considered significant. Data processing and statistical analyses were carried out with SPSS Statistics 22 software (IBM, Chicago, IL).

## RESULTS

No differences in patient characteristics were seen between the two groups (Table 1).

The number of seeds was significantly larger in the loose seed group than in the IBCL seed group; however, there was no significant difference in total activity between the two groups (Table 2). Although the same predetermined dosimetric parameters were used, D90 and D90 in the posterior base sector were significantly higher in the loose seed group than in the IBCL seed group in the planning phase. There was no significant difference in operation time between the two groups; however, operation time per seed was significantly longer in the IBCL seed group than in the loose seed group (1.31 min vs 1.13 min; *P* = 0.003).

**Table 2.** Planning phase

	IBCL group^a^	Loose group^a^	*P* value
*n*	32	32	
Prostate volume (ml)	31.4 ± 4.1	32.7 ± 4.8	0.27
No. of seeds	83.3 ± 7.0	89.2 ± 7.4	<0.001
No. of needles	23.9 ± 2.2	24.0 ± 1.8	0.81
Total activity (mCi)	30.6 ± 2.6	31.2 ± 2.6	0.18
D90 (Gy)	175.6 ± 6.7	179.6 ± 7.3	0.024
V100 (%)	99.4 ± 0.6	99.5 ± 0.7	0.96
V150 (%)	56.3 ± 6.3	59.6 ± 7.1	0.051
RV100 (ml)	0.40 ± 0.24	0.34 ± 0.22	0.38
RV150 (ml)	0.01 ± 0.02	0.01 ± 0.01	0.29
UD90 (Gy)	146.8 ± 8.5	151.7 ± 13.0	0.077
UD5 (Gy)	195.4 ± 13.8	194.2 ± 9.4	0.69
Prostate sector dosimetry			
D90 (Gy) Anterior base	175.8 ± 10.6	180.1 ± 11.7	0.13
Posterior base	177.7 ± 11.8	186.3 ± 12.1	0.005
Anterior mid-gland	185.2 ± 9.4	186.0 ± 10.4	0.76
Posterior mid-gland	179.3 ± 12.1	182.4 ± 8.4	0.23
Anterior apex	175.6 ± 8.8	176.5 ± 9.8	0.69
Posterior apex	161.8 ± 14.8	165.8 ± 12.8	0.26
V100 (%) Anterior base	99.5 ± 1.0	99.1 ± 1.7	0.33
Posterior base	99.7 ± 0.5	99.7 ± 0.5	0.26
Anterior mid-gland	99.9 ± 0.2	99.9 ± 0.3	0.57
Posterior mid-gland	99.4 ± 1.2	99.7 ± 0.4	0.93
Anterior apex	99.6 ± 0.7	99.5 ± 1.9	0.32
Posterior apex	96.3 ± 5.4	97.9 ± 2.3	0.80
Operation time (min)	108.7 ± 16.6	102.0 ± 15.2	0.13
Operation time/seed (min)	1.31 ± 0.21	1.13 ± 0.21	0.003

^a^Values are given as mean ± SD. D90 = the dose irradiating 90% of the prostate volume, V100 = the percentage of the prostate volume that receives 100% of the prescribed dose, V150 = the percentage of the prostate volume that receives 150% of the prescribed dose, RV100 = the rectal volume that receives 100% of the prescribed dose, RV150 = the rectal volume that receives 150% of the prescribed dose, UD90 = the dose irradiating 90% of the urethral volume, UD5 = the dose irradiating 5% of the urethral volume.

Table 3 shows the dosimetric results at 1 month and the number of patients with seed migration. In prostate sector dosimetry, V100 (95.3% vs 89.7%; *P* = 0.014) and D90 (169.7 Gy vs 152.6 Gy; *P* = 0.013) in the anterior base sector were significantly higher in the IBCL seed group than in the loose seed group. The seed migration rate was significantly lower in the IBCL seed group than in the loose seed group (6% vs 66%; *P* < 0.001).

**Table 3.** Post-implantation phase at 1 month

	IBCL group^a^	Loose group^a^	*P* value
*n*	32	32	
Prostate volume (ml)	29.0 ± 4.6	29.8 ± 4.0	0.47
D90 (Gy)	180.7 ± 12.7	178.1 ± 15.4	0.29
V100 (%)	98.2 ± 1.4	97.0 ± 2.4	0.057
V150 (%)	69.2 ± 9.9	68.8 ± 11.3	0.88
RV100 (ml)	0.97 ± 0.69	1.00 ± 0.71	0.78
RV150 (ml)	0.07 ± 0.09	0.11 ± 0.17	0.34
UD90 (Gy)	165.4 ± 19.4	154.6 ± 24.3	0.056
UD5 (Gy)	251.7 ± 28.3	246.4 ± 31.3	0.48
Prostate sector dosimetry			
D90 (Gy) Anterior base	169.7 ± 25.0	152.6 ± 28.3	0.013
Posterior base	180.1 ± 20.7	179.6 ± 23.1	0.93
Anterior mid-gland	196.5 ± 22.6	198.4 ± 27.9	0.77
Posterior mid-gland	189.9 ± 18.7	200.1 ± 27.1	0.08
Anterior apex	211.0 ± 26.1	198.3 ± 28.1	0.065
Posterior apex	180.1 ± 29.8	171.9 ± 24.8	0.23
V100 (%) Anterior base	95.3 ± 5.4	89.7 ± 10.1	0.014
Posterior base	98.3 ± 2.6	97.2 ± 5.9	0.35
Anterior mid-gland	99.2 ± 1.9	98.8 ± 2.8	0.86
Posterior mid-gland	99.4 ± 1.3	99.5 ± 1.3	0.54
Anterior apex	99.4 ± 1.6	99.0 ± 2.5	0.12
Posterior apex	95.8 ± 11.7	96.5 ± 6.0	0.39
Patient with seed migration	2	21	<0.001
Chest	1	15	
Abdominopelvic region	0	15	
Seminal vesicle	0	6	
Distal	1	1	

^a^Values are given as number (%) or mean ± SD. Abbreviations are as in Table 2.

Table 4 shows toxicities after brachytherapy. The median follow-up was 18 months (range, 1–36 months). No significant differences in toxicities were seen between the two groups.

**Table 4.** Crude rate of toxicity

	Grade	IBCL group^a^	Loose group^a^	*P* value
*n*		32	32	
GU	0	4 (13%)	4 (13%)	0.99
	1	27 (84%)	27 (84%)	
	2	1 (3%)	0 (0%)	
	3	0 (0%)	1 (3%)	
GI	0	30 (93%)	31 (94%)	0.56
	1	2 (6%)	1 (3%)	

^ a^Values are given as number (%). GI = gastrointestinal, GU = genitourinary.

## DISCUSSION

There are theoretical advantages in using linked seeds over using loose seed for PPB, including less seed migration and possible improved dosimetry if the implanted seeds do not migrate away from the prostate and stabilize due to linking [5, 10, 12]. Furthermore, the IBCL seeds system allows for combining seeds and connectors into seed trains of variable length and seed-to-seed spacing in the operating room, so it can meet the intraoperative planning method. The intraoperative planning method has a number of advantages over the preplanning method and some authors have reported it gives better dosimetric outcomes [13–15]. However, to date, there are only three studies that have compared IBCL with loose seeds in PPB [5, 9, 10]. Our analysis of 64 patients (32 treated with IBCL seeds and 32 treated with loose seeds) has shown that IBCL seeds provide more dose coverage post-operatively in the anterior base prostate sector and less seed migration than loose seed implantation.

Based on these results, we recommend that prostate cancer derived from the anterior fibromuscular stroma and transition zone be treated with IBCL seeds. Additionally, it is usually reasonable that seminal vesicle invasion suspected disease should be treated with IBCL seeds. Therefore, we believe that indications for the use of IBCL seeds include cases of prostate cancer derived from the anterior fibromuscular stroma and transition zone and cases in which seminal vesicle invasion is suspected. However, a higher dose in the anterior base sector may be correlated with a higher bladder neck dose, and might cause more urinary toxicity—Hathout *et al*. reported that, among the standard dosimetric variables, the dose to the bladder neck was the strongest predictor for acute and late urinary toxicity [16].

Table 5 shows the previous data comparing post-implant dosimetry between IBCL seeds and loose seeds. Although Jarusevicius *et al*. reported some DVH parameters differed significantly between the two groups [9], DVH parameters did not differ significantly in the other two reports [5, 10]. In our study, post-implant DVH parameters did not differ significantly between the two groups, consistent with most prior data.

**Table 5.** Previously published data comparing post-implant dosimetry between IBCL seeds and loose seeds

Year	[Ref]	*n*	DVH parameter	IBCL seed^a^	Loose seed^a^	*P* value
2010	[5]	91	D90 (Gy)	Pd: 104.0	Pd: 98.2	0.42
				I: 165.1	I: 164.5	0.36
			% of RV100 > 1.3 ml	16.7	23.3	NS
2012	[9]	230	D90 (Gy)	177.9	184.7	0.002
			V100 (%)	94.9	95.5	0.21
			UD30 (Gy)	197.4	218.6	0.001
			RV100 (ml)	0.3	0.6	<0.001
2014	[10]	140	D90 (Gy)	174.4	170.7	NS
			V100 (%)	96.6	95.7	NS
			UD30 (Gy)	203.2	206.8	NS
			RV100 (ml)	0.47	0.51	NS
Present study		64	D90 (Gy)	180.7	178.1	0.29
			V100 (%)	98.2	97.0	0.057
			UD5 (Gy)	251.7	246.4	0.48
			RV100 (ml)	0.97	1.00	0.78

^a^Values are given as mean or %. DVH = dose–volume histogram, UD30 = the dose irradiating 30% of the urethral volume. Other abbreviations are as in Table 2.

Regarding prostate sector dosimetry, many studies have reported that the anterior base sector and the base sector received a lower dose [8, 17–21]. Seed migration, needle drag, needle splay, the proximity of the prostate base to the bladder, and the difficulty in accurately contouring the prostate base are proposed as possible explanations for the lower dose in the anterior base and base sectors [17–21]. Seed migration is thought to be the reason why the dose in the anterior base sector decreased more in the loose seed group than in the IBCL seed group in our study. Because of the proximity of the venous plexus to the anterior base sector, it is reasonable to speculate that the majority of migrating seeds were intended for implant in the anterior base sector [17, 19]. Indeed, in our study, V100 (88.1% vs 92.4%; *P* = 0.043) and D90 (145.5 Gy vs 164.4 Gy; *P* = 0.068) in the anterior base sector were lower in the loose seed patients with seed migration into the chest and/or abdominopelvis (*n* = 20) than the loose seed patients without it (*n* = 12). Therefore, the dose in the anterior base sector might have decreased more in the loose seed group due to more seed migration than in the IBCL seed group.

Ishiyama *et al*. reported that the seed migration rate was significantly lower in the IBCL seed group (0%) than in the loose seed group (55%; *P* < 0.001) [10]. Several other studies have also reported that the seed migration rate was significantly lower in the stranded seed group than in the loose seed group [12, 22, 23]. Our study had similar result to theirs.

Zauls *et al*. [5] and Ishiyama *et al*. [10] reported the operation time was significantly longer in the IBCL seed group than in the loose seed group. In both their reports, there was no significant difference in the number of seeds between the IBCL seed group and the loose seed group. In the present study, there was no significant difference in operation time between the IBCL seed group and the loose seed group. However, the number of seeds was significantly larger in the loose seed group than in the IBCL seed group, and the operation time per seed was significantly longer in the IBCL seed group than in the loose seed group. We believe this was because we were accustomed to loose seed brachytherapy and constructing and placing IBCL seeds took a longer time than placing loose seeds.

The number of seeds was significantly larger in the loose seed group than in the IBCL seed group in our study. However, no significant differences were seen in prostate volume and total activity between the two groups, probably because the activity of the loose seed was 0.35 mCi/seed and that of the IBCL seed was 0.367 mCi/seed. Our study showed that D90 and D90 in the posterior base sector were significantly higher in the loose seed group than in the IBCL seed group in the planning phase. This may be attributable to unintentional bias. For the IBCL seed, we placed a few seeds inside the seminal vesicle, if necessary; therefore, we seem to have placed more seeds inside the prostate capsule in the posterior base sector for the loose seed to achieve V100 > 99% than for the IBCL seed. This may have contributed to the differences in D90 and D90 in the posterior base sector.

Our study has some limitations. This is a retrospective study. However, all patients were treated by the same radiation oncologist (N.K.) and urologist (A.T.). Other than placing a few seeds inside the seminal vesicle, if necessary, in the IBCL seed group, all other procedures and dose–volume targets remained the same between the two groups. No differences in patient characteristics were seen between the two groups. It is possible that intra-observer variability in post-implant dosimetry occurred [24]. However, CT/MRI fusion, which we used, is regarded as the best method for post-implant dosimetry [25–27]. The radiation oncologist (N.K.) had performed more than 100 post-implant dosimetries using CT/MRI fusion before our study. Therefore, intra-observer variability in post-implant dosimetry should be limited in our study.

In conclusion, our study showed more dose coverage postoperatively in the anterior base prostate sector and less seed migration in IBCL seed implantation than in loose seed implantation.

## CONFLICT OF INTEREST

Yasutomo Nasu owns the stock of Momotaro-Gene Inc., a company relevant to prostate cancer gene therapy, outside the submitted work.
